# The extant World War 1 dysentery bacillus NCTC1: a genomic analysis

**DOI:** 10.1016/S0140-6736(14)61789-X

**Published:** 2014-11

**Authors:** Kate S Baker, Alison E Mather, Hannah McGregor, Paul Coupland, Gemma C Langridge, Martin Day, Ana Deheer-Graham, Julian Parkhill, Julie E Russell, Nicholas R Thomson

**Affiliations:** a Wellcome Trust Sanger Institute, Wellcome Trust Genome Campus, Hinxton, Cambridgeshire, UK; b National Collection of Type Cultures, Public Health England, Porton Down, Salisbury, UK; c Gastrointestinal Bacteria Reference Unit, Public Health England, London, UK; d Department of Infectious and Tropical Diseases, London School of Hygiene & Tropical Medicine, London, UK

## Abstract

**Background:**

Shigellosis (previously bacillary dysentery) was the primary diarrhoeal disease of World War 1, but outbreaks still occur in military operations, and shigellosis causes hundreds of thousands of deaths per year in developing nations. We aimed to generate a high-quality reference genome of the historical *Shigella flexneri* isolate NCTC1 and to examine the isolate for resistance to antimicrobials.

**Methods:**

In this genomic analysis, we sequenced the oldest extant *Shigella flexneri* serotype 2a isolate using single-molecule real-time (SMRT) sequencing technology. Isolated from a soldier with dysentery from the British forces fighting on the Western Front in World War 1, this bacterium, NCTC1, was the first isolate accessioned into the National Collection of Type Cultures. We created a reference sequence for NCTC1, investigated the isolate for antimicrobial resistance, and undertook comparative genetics with *S flexneri* reference strains isolated during the 100 years since World War 1.

**Findings:**

We discovered that NCTC1 belonged to a 2a lineage of *S flexneri,* with which it shares common characteristics and a large core genome. NCTC1 was resistant to penicillin and erythromycin, and contained a complement of chromosomal antimicrobial resistance genes similar to that of more recent isolates. Genomic islands gained in the *S flexneri* 2a lineage over time were predominately associated with additional antimicrobial resistances, virulence, and serotype conversion.

**Interpretation:**

This *S flexneri* 2a lineage is a well adapted pathogen that has continued to respond to selective pressures. We have created a valuable historical benchmark for shigellae in the form of a high-quality reference sequence for a publicly available isolate.

**Funding:**

The Wellcome Trust.

## Introduction

*Shigella* spp are Gram-negative bacteria that cause shigellosis (previously known as bacillary dysentery)—a severe enteric illness transmitted faeco-orally via a very low infectious dose.^1^ The highly toxigenic species *Shigella dysenteriae* was first described in 1898, followed rapidly by *Shigella flexneri* in 1900.^2^, ^3^ Only 14 years later, shigellae became infamous as the main diarrhoeal agents in World War 1,^4^ and today continue to cause outbreaks during military campaigns.^5^, ^6^ Diarrhoeal disease kills about 750 000 children younger than 5 years annually,^7^ and shigellae are one of the top four causes of moderate-to-severe diarrhoea in this age group in developing nations.^8^ In these countries, which carry most of the shigellosis burden, most cases of disease (745 [66%] of 1130 cases according to one study^9^) are caused by *S flexneri*, with serotype 2a being highly prevalent. Further study of the biological mechanisms underpinning the success of *Shigella* as a pathogen and how it continues to evolve is still needed.

By contrast with microbiologists approaching this question during World War 1, today's investigators can make use of advances in molecular methods to analyse mechanisms of virulence in detail by sequencing pathogen genomes. *Shigella* are specialised clades of *Escherichia coli* adapted to human beings, characterised by having additional elements in their genome that produce heightened virulence. In addition to having a large virulence plasmid for the invasion of intestinal cells,^10^ *Shigella* spp contain large numbers of insertion sequence (IS) elements relative to other enteric bacteria.^11^ These elements help with the horizontal transfer of genomic information and are responsible for the mobility of many pathogenic determinants in *Shigella*.^12^ Studies of the horizontal evolution of the *Shigella* chromosome—which is key to virulence—are difficult because the length (up to 2700 bp) and high number of perfectly repeated IS elements interfere with genome assembly when using sequence data generated by short-read technologies.^13^ This limitation has restricted whole-genome sequencing work on *Shigella*,^14^, ^15^, ^16^ providing limited insight into horizontal gene transfer in the chromosome. Overcoming this issue of genome reconstruction to study complete reference genomes would help the investigation of horizontal evolution.

Compared with the microbiologists of World War 1, investigators now have the benefit of 100 years of hindsight. The study of pathogen genomes over a long timecourse can inform epidemiology and help to identify the constant and inconstant elements that are necessary for pathogen survival or contributory to its success.^17^, ^18^ In this study, we present the genome sequence and comparative genomics of the oldest extant *S flexneri* isolate, NCTC1. This strain was isolated in 1915 from an early case of dysentery reported in the British forces on the Western front in World War 1.^19^

In 1920, NCTC1 and other *S flexneri* types were the first bacteria accessioned into the National Collection of Type Cultures (NCTC),^20^ the longest-running collection of human pathogenic bacteria in the world. The microbiologists responsible for these isolates worked during revolutions of microbiological theory, sharing their lifetimes with Robert Koch and Louis Pasteur. During World War 1, strains of dystentery were brought together from all over the world, facilitating the collection of a series of representative strains. Microbiologists aimed to preserve this unprecedented diversity of *S flexneri* in the hope that “more rapid understanding could be reached…by more intensive study of a centralized collection”.^20^ 100 years later, microbiology has been similarly transformed by molecular biology and genomic techniques. To build on the foresight of those early, enlightened microbiologists, we applied the methods of the current microbiological revolution to sequence the preserved strain.

We generated a high-quality reference genome for NCTC1 using single-molecule real-time (SMRT) technology and examined the isolate for resistance to antimicrobials. The genome of NCTC1 creates a historical context for the study of the evolution of shigellae, shown here by comparative genetics of a timecourse of other strains from this epidemiologically important lineage isolated in the 100 years since World War 1.

## Methods

### Storage and laboratory manipulation of NCTC1

Few records exist that describe the storage of NCTC1 before 1935 in detail. However, archived documents suggest that it was subcultured from stock cultures on Dorset's egg medium that were several years old. For this study, NCTC1 stock (freeze dried since 1951) was grown on 5% blood agar at 37°C for 18–24 h in aerobic conditions and a 10 uL loop-full of growth harvested onto cryoprotective beads (Protect Multipurpose, Technical Service Consultants Ltd, Lancashire, UK) and stored at –80°C. To obtain DNA, a 1 uL loop-full of thawed cells was lysed (750 uL of lysis buffer; Promega, Madison, WI, USA) at 80°C for 15 min (5 uL of RNAse was also added) and frozen at –80°C. We used thawed lysate for DNA extraction using the Promega Maxwell 16 unit (Maxwell 16 Cell DNA Purification Kit). The presence of DNA was confirmed using the Qubit fluorometer (Life Technologies, Paisley, UK).

We tested NCTC1 for resistance against a range of modern antimicrobials using an agar (Mueller-Hinton and Iso-Sensitest) dilution method. The antimicrobials and concentrations tested were: chloramphenicol (8 mg/L and 16 mg/L), colomycin (2 mg/L), sulphonamide (256 mg/L), gentamicin (2 mg/L), tobramycin (8 mg/L), amikacin (8 mg/L), streptomycin (8 and 16 mg/L), tetracycline (8 mg/L), trimethoprim (2 mg/L), nalidixic acid (16 mg/L), ciprofloxacin (0·064 mg/L and 0·5 mg/L), cefoxitin (8 mg/L), ceftazidime (1 mg/L and 2 mg/L), cefotaxime (0·5 mg/L and 1 mg/L), cefpirome (1 mg/L), ertapenem (0·064 mg/L and 0·5 mg/L), and temocillin (128 mg/L). Minimum inhibitory concentrations (MICs) against clemizole penicillin, erythromycin, and ampicillin were established using Etest strips.

### Genome data

We generated a PacBio SMRT sequencing library from 2 μg genomic DNA sheared to about 20 kb with a 26 G blunt Luer-lok needle and sequenced it on a Pacific Biosciences RSII DNA sequencing system (Manlo Park, CA, USA). Library preparation was according to the manufacturer's recommendation, with an additional bead clean-up (with a ratio of 0·375 ×) before primer annealing. The library was bound with P4 polymerase and complexes were loaded on to version V3 SMRT cells. These were sequenced using 180 min movies (using version C2 chemistry), generating 555 581 individual reads of the DNA fragments (known as subreads) with an N50 of 3·1 kb. De-novo assembly of these reads was done with HGAP.3 (Pacific Biosciences), on the SMRT Analysis pipeline version 2.2.0. The NCTC1 chromosome was recovered as a contiguous sequence of 4 535 149 bp with about 250× coverage. Circularisation was achieved by manual comparison and removal of a region of overlap, and we confirmed the final genome by remapping of sequence data.

An Illumina library with a rough insert size of 450 bp (range 177–1241) was generated and sequenced (Illumina MiSeq, San Diego, CA, USA) according to in-house protocols.^21^ Assembly of the 2 309 646 sequencing reads (European Nucleotide Archive accession number ERS428535) from the paired end in a 150 cycle (bp) was run using the VelvetOptimiser script.^22^

### Bioinformatic analysis

We compared NCTC1 genome data generated in this study with reference genomes of *S flexneri, S dysenteriae, Shigella sonnei, Shigella boydii,* and *E coli,* as well as draft genomes of *S flexneri* serotyping strains held by Public Health England (PHE). Comparative genomics was facilitated by annotation of the NCTC1 chromosome and re-annotation of existing *S flexneri* reference chromosomes by the software tool Prokka.^23^ We used annotations to identify insertion sequences and define core and accessory genomes. We identified orthologous protein groups by iterative clustering using the CD-HIT (Cluster Database at High Identity with Tolerance) program (≥90% identity) and BLAST (Basic Local Alignment Search Tool) analysis (98% identity threshold) and inflated them by further clustering using the Markov cluster algorithm.^24^, ^25^, ^26^ The core genome consisted of orthologous protein groups present once in all genomes, and the accessory genome consisted of those protein groups that were absent in one or more genomes. We identified accessory genome regions (or islands) with more than two consecutive orthologous protein groups and examined them for function and presence in reference genomes. We detected protein orthologues of antimicrobial resistance genes (ARGs) using the ARDBAnno program^27^ and manually examined proteins with quinolone-resistance-associated mutations.

We identified coordinates and generated image files using ABACAS (Algorithm-Based Automatic Contiguation of Assembled Sequences), Artemis, the Artemis Comparison Tool,^28^ and DNAPlotter. For phylogenetic analysis, we generated a multiple sequence alignment by mapping Illumina sequencing data (or in-silico generated fastq files) to the NCTC1 chromosome using SMALT software^29^ and removal of regions of recombination.^30^ We then used the variable sites (147 943 bp) of the alignment to construct a maximum likelihood tree using RAxML 7.8.6.^31^

### Role of the funding source

The funder of the study had no role in study design, data collection, data analysis, data interpretation, or writing of the report. The corresponding author had full access to all the data in the study, and had final responsibility for the decision to submit for publication.

## Results

We sequenced NCTC1 using long-read (SMRT) sequencing technology, and constructed the complete, circularised 4 526 576 bp genome from a single contiguous sequence of about 250× coverage (European Bioinformatics Institute accession number LM651928). We used data from short-read (Illumina) sequencing to investigate the complications of genome assembly using this technology, and to validate the genome further. The short-read sequence data was assembled into a draft genome of 4 301 901 bp (272 contiguous sequences, N50 37 887 bp), in which contiguous sequences (the breaks in which were frequently associated with IS elements) were distributed throughout the NCTC1 genome (appendix). We detected no single nucleotide polymorphisms (SNPs) when the short-read sequence data were mapped to the completed genome.

**Figure 1:**
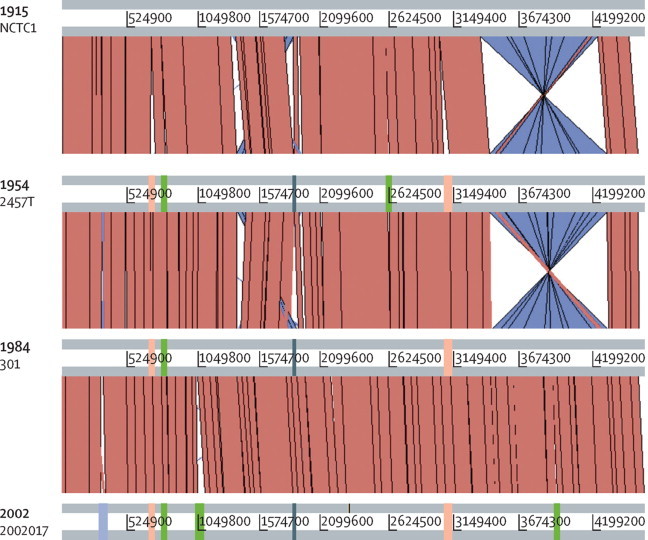
Comparative genomics of *Shigella flexneri* reference genomes in 2a lineage The genomes are depicted as horizontal grey lines lines interspersed with regions of colinear (red) and inverted (purple-blue) synteny. Genomes are arranged from top to bottom by year of isolation. Comparative gain of genomic islands is shown by blocks overlying genomes, and are coloured by functional association of antimicrobial resistance (green), virulence (peach), serotype conversion (light purple), and other (dark blue-grey).

To investigate similarities with other *S flexneri* genomes, we identified the basic characteristics and genome features of NCTC1. The 4 526 576 bp genome with 4564 coding sequences was slightly (1·6–2·7%) smaller than existing reference sequences (table 1), but like many *S flexneri,* NCTC1 had a guanine–cytosine content of 51% (2 308 554 of 4 526 576 bp). The guanine–cytosine content and guanine–cytosine skew varied along the genome, which could be a result of the highly dynamic system of rearrangements and horizontal acquisitions, most of which are conserved in more recent *S flexneri* isolate genomes (appendix). NCTC1 carried the phage-borne *gtrII* gene responsible for conferring the 2a serotype,^32^ but we recovered no sequence data derived from the large virulence plasmid (or other plasmids). The genome of NCTC1 was similar in arrangement to other *S flexneri* (figure 1), with the exception of a large inversion and double recombination relative to the 2457T strain (previously reported for the 301 strain).^33^ It shared a large (3919 genes) core genome with *S flexneri* isolates from the 2a lineage (table 1), 3704 of which were also found in the *S flexneri* serotype 5 genomes (M90T and 8401; data not shown). Similar to other *S flexneri* genomes, NCTC1 contained 391 coding sequences related to IS elements (table 1) that covered 6% (271 595 of 4 526 576 bp) of the total genome, of which IS1 and IS600 were the most common (appendix).

**Table 1:** Isolate and genome characteristics of reference genomes in the *Shigella flexneri* 2a lineage compared with NCTC1

	**Serotype**	**Year isolated**	**Location of isolation**	**Length, bp**	**Associated plasmids**	Coding sequences annotated*		**Comparison with NCTC1**	
						Unique† (total)	IS element	SNPs	Shared unique
NCTC1	2a	1915	France	4 526 576	0	4058 (4532)	391	0	4058
2457T	2a	1954	Japan	4 599 354	4	4150 (4617)	401	457	4022
301	2a	1984	China	4 607 202	1	4123 (4602)	382	719	4034
2002017	Xv	2002	China	4 650 856	5	4196 (4681)	400	531	3982

The total number of unique coding sequences present across the four isolates was 4344, of which 3919 were shared by (present in all) isolates. IS=insertion sequence. SNP=single nucleotide polymorphism.

* Annotation results from this study.

† Refers to orthologous protein groups.

**Figure 2:**
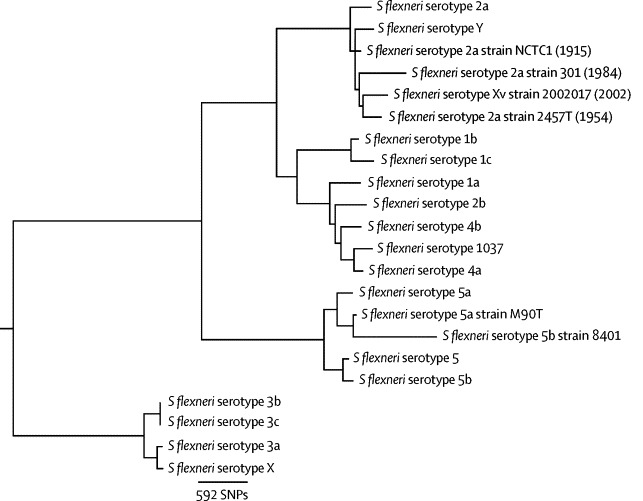
Evolutionary relationships of *Shigella flexneri* The phylogenetic tree shows the evolutionary relations among reference genomes and draft genomes of serotyping reference isolates held at Public Health England. Four completed genomes (followed by year of isolation) that cluster within the serotype 2a lineage are shown in grey. All bootstrap values were 96 or greater and mapping coverage of draft genomes and simulated fastq data from reference genomes were equivocal.

Phylogenetic analysis of mutational differences was used to place NCTC1 in a *S flexneri* 2a lineage. Relative to NCTC1, the five completed reference genomes had between 457 and 719 SNPs (within 2a lineage, table 1) and 3266 and 3608 SNPs (serotype 5 references M90T and 8401 respectively; figure 2). We used other *Shigella* species and *E coli* genomes, and draft genomes of the *S flexneri* serotyping strains held at PHE, to establish the phylogenetic relations between a broader set of isolates. This showed that NCTC1 clustered phylogenetically with isolates of serotypes 2a, Y, and Xv (figure 2), in an established genetic context in which *S flexneri* serotypes 1–5 are virtually monophyletic within the *Shigella* and *Escherichia* genera^34^ (appendix).

To examine changes in the *S flexneri* 2a lineage accessory genome over time, we identified discrepancies in chromosomal gene content among complete reference sequences (we excluded draft genome assemblies because plasmid and chromosomal material was indistinguishable). These genomes were the common laboratory strain 2457T, isolated in Japan in 1954; 301, isolated in Beijing in 1984; and 2002017, a 2002 isolate of the *S flexneri* serotype Xv that is epidemic in China^35^ (grey highlight in figure 2; table 1). Only 14 genomic islands differed among these chromosomes, and these were mainly associated with antimicrobial resistance, virulence, and serotype conversion (appendix). NCTC1 contained virtually no new genetic information; the only unique genomic island in NCTC1 was a cluster of diguanylate-cyclase pseudogenes (table 1; appendix). Four genomic islands distributed throughout the genome were gained ubiquitously in the more modern isolates (figure 1). These genomic islands were the previously characterised *ipaH*-island I and *she*PAI (SHI-1) island, an uncharacterised island encoding the antimicrobial resistance gene (ARG) *mdfA* and a peptidoglycan biosynthesis gene, and an uncharacterised island carrying genes related to aminoacid metabolism and an inner membrane ABC transporter (figure 1). Similarly, three genomic islands were uniquely present in the most modern isolate (2002017), a serotype conversion island, and two antimicrobial resistance determinants (figure 1; reported elsewhere^35^). The only other genomic island gained in time relative to NCTC1 was an island containing resistance genes to sulphonamide antibiotics (*sul2*) and other organic and inorganic chemicals in isolate 2457T (figure 1; appendix). The remaining five islands represent losses over time and were mainly uncharacterised (with the exception of SfII lost from 2002017; appendix). Many previously characterised islands of shigellae, including *sci, ipaH* islands II, III, IV, V, and SHI-2, were found in all isolates.

**Figure 3:**
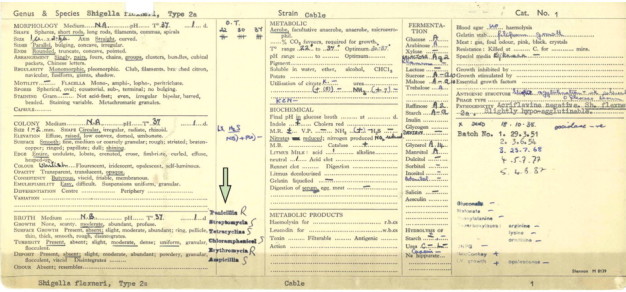
Antimicrobial resistance in NCTC1 The accession card for NCTC1 notes antimicrobial resistance phenotyping (arrow) apparently done in the 1950s. Please note, NCTC1 is incorrectly noted as an aerobe here (all shigellae are facultative anaerobes).

We examined the phenotype and genome of NCTC1 for resistance to antimicrobials. The NCTC1 accession card reported resistance to erythromycin and penicillin in tests done in the 1950s (figure 3). These phenotypes were confirmed as part of this study, with NCTC1 having MICs of 16 mg/L and 24 mg/L for these compounds respectively. NCTC1 had a MIC of 1 mg/L against ampicillin, and was also phenotypically sensitive to breakpoint concentrations of all the other antimicrobials we tested. We established the ARG content of the reference genomes in the *S flexneri* 2a lineage (table 2), and identified ARGs consistent with the observed NCTC1 phenotype. These were a tripartite efflux pump (*mdtE, mdtF*, and *tolC)* capable of conferring resistance to erythromycin and a β-lactamase (*bl1_ec*) that could confer resistance to penicillin. The β-lactamase orthologue was an *ampC* gene that is widely conserved throughout many *Enterobacteriacae*.^36^ The chromosomal ARG complement of the three strains isolated between 1915 and 1984 were similar, whereas the isolate from 2002 contained more ARGs (table 2).

**Table 2:** Locus tags of chromosomal antimicrobial resistance gene orthologues in reference sequences from the *S flexneri* 2a lineage

	**NCTC1**	**2457T**	**301**	**2002017**
*acrA*	NCTC1_00422	S0415	SF0408	SFxv_0453
*acrB*	NCTC1_00421	S0414	SF0407	SFxv_0452
*arnA*	NCTC1_02567	S2467	SF2334	SFXv_2577
*bacA*	NCTC1_03355	S3303	SF3098	SFXv_3402
*bcr*	NCTC1_02492	S2398	SF2269	SFXv_2504
*bl1_ec*	NCTC1_04677	S4573	SF4308	SFXv_4697
*emrE*	NCTC1_02153	S4821	SF1980	SFXv_2210
*ksgA*	NCTC1_00048	S0050	SF0048	SFxv_0050
*macB*	NCTC1_00837	S0879	SF0839	SFxv_0909
*mdfA*	..	S0838	SF0795	SFxv_0864
*mdtE*	NCTC1_03830, NCTC1_03893	S4169	SF3599, SF3547	SFXv_3923, SFXv_3866
*mdtF*	NCTC1_03891, NCTC1_03892	..	SF3598, SF3597	SFXv_3922
*mdtG*	NCTC1_01107	..	..	SFxv_1206a
*mdtH*	NCTC1_01504	S1149	SF1071	SFxv_1219
*mdtK*	NCTC1_01835	S1823	SF1691	SFxv_1899
*mdtL*	NCTC1_04048	S4019	SF3752	SFXv_4079
*mdtN*	NCTC1_04506	S3608	SF4142	SFXv_4519
*mdtO*	NCTC1_04505	S2587	SF4141	SFXv_4518
*mdtP*	NCTC1_04504	S3607	SF4140	SFXv_4517
*tolC*	NCTC1_03331	S3280	SF3075	SFXv_3376
*sul2*	..	S2703	..	..
*ant3iA*	..	..	..	SFXv_4143, SFXv_1143
*bl2d_oxa1*	..	..	..	SFXv_1144
*catA1*	..	..	..	SFXv_1150
*dfrA1*	..	..	..	SFXv_4142a
*tetB*	..	..	..	SFXv_1153, SFXv_1140

All isolates had wild type *gyrA/gyrB/parC/parE* gene sequences.

## Discussion

The genome of NCTC1, isolated during World War 1, provides a benchmark for evolutionary studies of shigellae, as shown here. Factors now known to have played a part in the dissemination of *Shigella* spp during World War 1 can be contrasted with those of the present day to assist the discussion of the significance of this study's findings. A retrospective analysis of dysentery in German forces during World War 1 identified four main reasons for the uncontrollable outbreaks: poor hygiene; predisposition through malnutrition; problems with bacteriological diagnosis; and absence of specific therapies.^4^ Although the first two explain the current epidemiological distribution of shigellosis as a disease mainly of children in developing nations, the results of studies such as this, which used advanced bacterial diagnosis—ie, whole genome sequencing—might provide information that could help investigators to develop specific therapies.

Phylogenetic analysis showed that NCTC1 was part of a *S flexneri* 2a lineage, and comparison with reference genomes in this lineage showed the genome to be relatively stable over time. The NCTC1 chromosome was much the same in terms of composition (eg, length, guanine–cytosine content) and content, with a similar number of insertion sequences, and 98% of genes (3982 of 4058) from NCTC1 being preserved in modern isolates. However, we could not do a comparative study of plasmids because we recovered no plasmids from NCTC1. The large virulence plasmid was probably lost through serial passage (as previously reported^37^), which suggests that NCTC1 might have contained other plasmids, as seen in other enteric pathogens of the pre-antibiotic era.^38^ In these ways, the *S flexneri* of 100 years ago was already a highly derived lineage well adapted to its niche.

Another way in which *S flexneri* was set to persist was the presence of intrinsic antimicrobial resistance. NCTC1 was isolated in 1915, before the description of penicillin in 1929^39^ and its widespread clinical use. However, NCTC1 was resistant to penicillin and erythromycin. The presence of ARGs in NCTC1 is consistent with the ancient origins of antimicrobials for other ecological purposes (eg, quorum sensing and competition factors),^40^ and signified the potential in *S flexneri* for rapid development of further resistance. For example, orthologues of the NCTC1 *ampC* gene need only small mutations in the promoter region to confer high-level resistance against contemporary β-lactam antibiotics.^41^ Intrinsic ARGs on the NCTC1 chromosome, which contain a similar complement of chromosomal ARGs to *S flexneri* isolated as late as 1984, show how shigellae were well placed to meet the challenge presented by the subsequent widespread use of antimicrobials even in 1915.

Although NCTC1 was sensitive to a panel of modern antimicrobials, changes in the *S flexneri* lineage 2a chromosome over time show how shigellae are likely to have adapted to evolutionary pressures. Over nearly 100 years, the *S flexneri* 2a lineage showed the relative gain (either through selection of pre-existing diversity or acquisition and subsequent evolutionary success) of eight genomic islands. Half these islands conferred resistance to antimicrobials and other chemical compounds and a further three were related to virulence (the enterotoxin encoding *she*PAI^42^ and *ipaH* island 1^11^) and serotype conversion. Serotype conversion might theoretically lead to immune evasion, because immunity to *S flexneri* is serotype specific.^43^ The apparent accumulation of virulence, serotype conversion, and antimicrobial resistance determinants (an observation that might have been enhanced if plasmids had been available for examination) exemplifies how the 2a lineage has adapted to evolutionary pressures, and is consistent with the appearance of multidrug and extended-spectrum antimicrobial-resistant shigellae.^44^, ^45^ In the face of this increasing drug resistance, soon no specific therapies for shigellae will be available.

Panel **Research in context**

**Systematic review**

We searched public nucleotide databases (National Centre for Biotechnology Information, European Nucleotide Archive) for completed *Shigella flexneri* genome sequences. We downloaded and reviewed the sequences and the associated articles for isolation information. In addition to an existing library of articles on shigellae evolution and bacteriology, we used the following specific scientific literature searches in PubMed and Scopus (war AND [shigell* OR bacillary dysentery]) and (ampC AND shigell*) and downloaded and reviewed full articles. We searched culture collections (National Collection of Type Cultures, and American Type Culture Collection) for *S flexneri* isolates associated with dates. We also reviewed the accession card and information relating to NCTC1 on the National Collection of Type Cultures (NCTC) website and in archives held at Porton Down, UK.

**Interpretation**

This study contributes a high-quality reference genome of the oldest extant *S flexneri* isolate from a case of dysentery in World War 1. We show that this 1915 isolate, from before the discovery of penicillin, has intrinsic resistance to antimicrobials. We show the usefulness of SMRT-technology sequencing for overcoming the repetitive nature of these genomes. By using this isolate as a historical backdrop and comparing it with isolates from the 100 years since World War 1, we present evidence that the *S flexneri* 2a genome is gaining drug resistance, virulence, and serotype conversion islands over time. The sequenced isolate is publically available from the NCTC under NCTC1.

Despite the availability of only three complete genomes of the 2a lineage, by sequencing the NCTC1 isolate we have provided a historical backdrop for the evolution of *S flexneri* that has enabled us to reflect on the factors that have contributed to the continued dissemination of shigellosis since World War 1. Advances in bacteriological diagnosis have been made and results from the application of the technology presented here suggest that prevention is the best approach to control shigellae in view of their intrinsic antimicrobial resistance and targeted evolution. Continued efforts to improve hygiene and malnutrition in developing countries will have a substantial effect but these efforts are impeded by the low infectious dose of shigellae. More than anything, specific therapies for *Shigella*, and particularly a licensed *Shigella* vaccine, are urgently needed.

## Supplementary Material

Supplementary appendix

Supplementary video Genetic analysis of an almost 100-year-old strain of *Shigella* isolated from a World War 1 soldier plots the changes in the *Shigella* genome over time.
