# Supplementary material for: The extant World War 1 dysentery bacillus NCTC1: a genomic analysis
Source: Lancet. 2014 Nov;384(9955):1691–7. doi: 10.1016/S0140-6736(14)61789-X (PMC4226921; doi:10.1016/S0140-6736(14)61789-X)
Supplement: Supplementary appendix [file mmc1.pdf]

THE LANCET

Supplementary appendix

This appendix formed part of the original submission and has been peer reviewed. We post it as supplied by the authors.

Supplement to: Baker KS, Mather AE, McGregor H, et al. The extant World War 1 dysentery bacillus NCTC1: a genomic analysis. *Lancet* 2014; **384:** 1691–97.

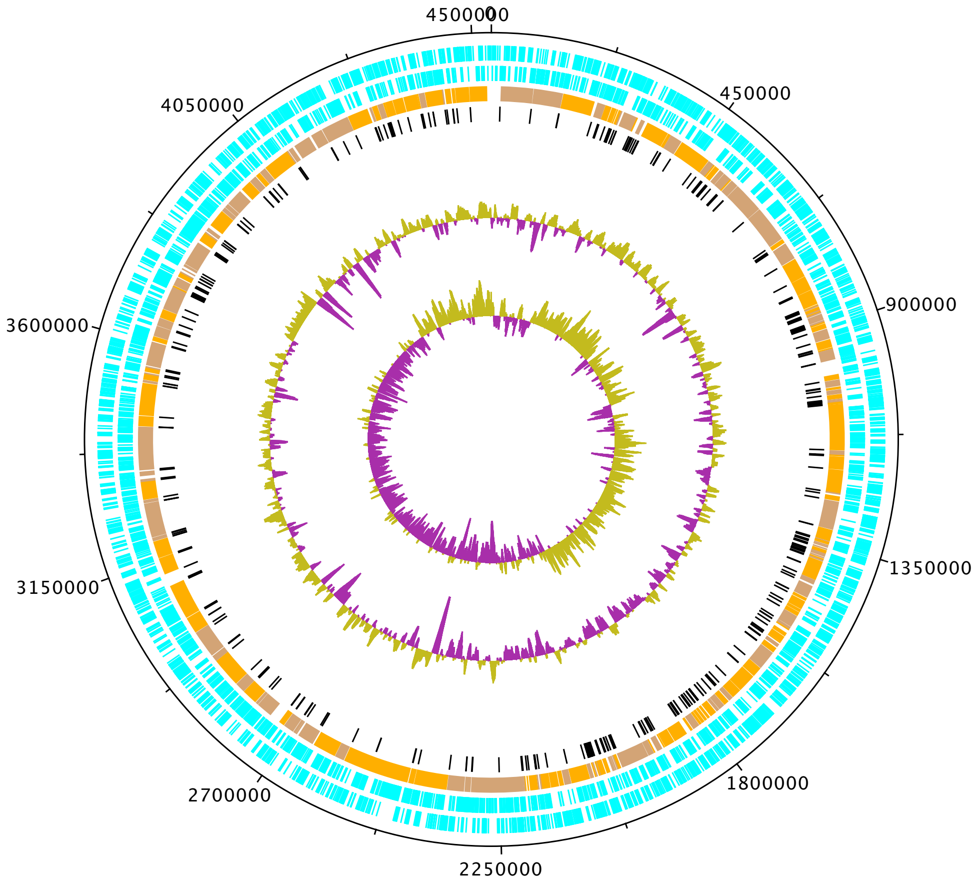

The NCTC1 genome and draft genome. The NCTC1 genome is represented circularly with the two outer tracks showing annotation coordinates of the forward and reverse strands. The third track shows coordinates of contiguous sequences generated in the draft genome, often bound by insertion sequences (shown in black on the fourth track). The GC content is shown as the fifth track and the GC skew is innermost.

Number of annotations related to IS elements in NCTC1

IS element	No. annotations	IS CDSs (number)
IS1	125	2
IS150	13	2
IS2	71	2
IS294	4	1
IS3	7	2
IS4	21	2
IS600	56	2
IS629	15	2
IS91	8	1
IS911	34	2
ISEhe	6	2
Iso-IS1	2	2
ISSfl3	10	1
ISSfl4	19	3

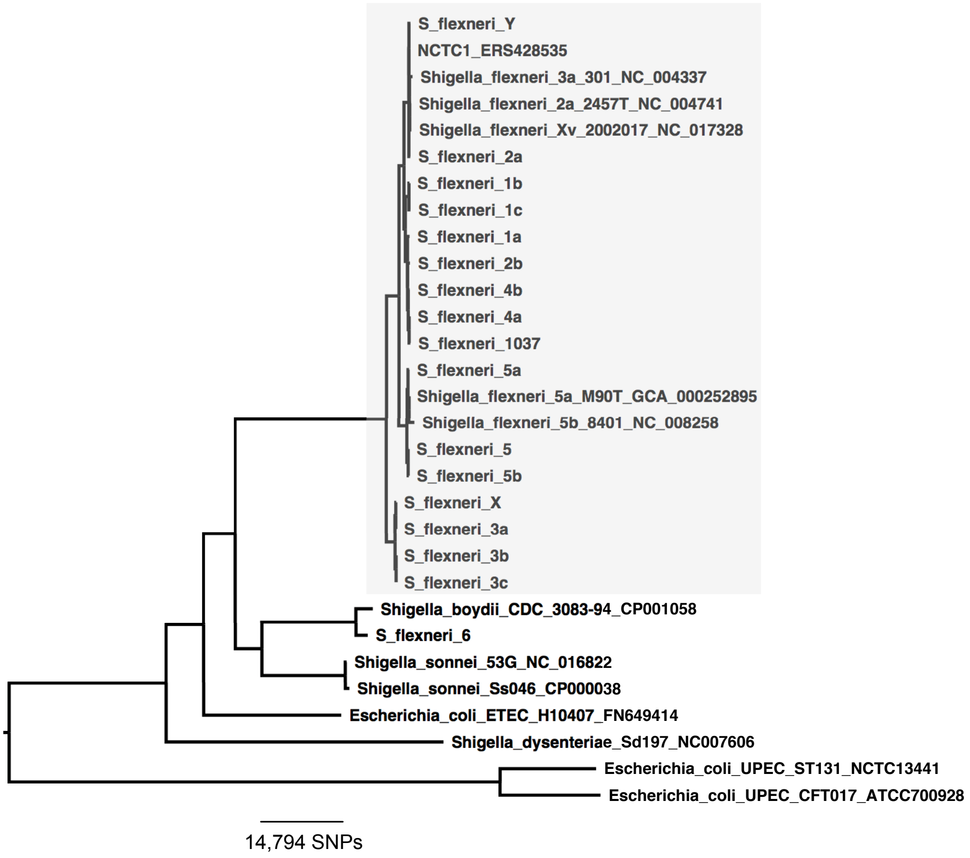

The phylogenetic context of Shigella flexneri. The portion of the tree that is shown in Figure 2 is highlighted in grey. The names of reference genomes are followed by their accession numbers in the following databases: European Nucleotide Archive, National Center for Biotechnology Information, American Type Culture Collection, or National Collection Type Cultures. All bootstrap values were at least 96.

Genomic islands identified as dynamic among chromosomal sequences of the *S. flexneri* 2a lineage

		Reference genomes				Genomic position	
**Genomic island**	**Function**	**NCTC1**	**2457T**	**301**	**2002017**	**Positions**	**Genome**
SHI-O	Serotype conversion	Absent	Absent	Absent	Present	310331..348225	2002017
SfII (serotyping island)	Serotype conversion	Present	Present	Present	Absent	311757..340670	301
ipaH island 1	Virulence	Absent	Present	Present	Present	714172..749781	301
Uncharacterised island (*mdfA*)	Drug resistance	Absent	Present	Present	Present	823260..832507	2457T
SRL-like pathogenicity island	Drug resistance	Absent	Absent	Absent	Present	1081929..1130024	2002017
Uncharacterised island	Metabolism	Absent	Present	Present	Present	1841204..1852440	2457T
Uncharacterised island (*sul2*)	Drug resistance	Absent	Present	Absent	Absent	2598561..2613997	2457T
SHI-1 (*she*PAI)	Virulence	Absent	Present	Present	Present	3054015..3104758	301
Tn7-like element	Drug resistance	Absent	Absent	Absent	Present	3944748..3958815	2002017
Uncharacterised island	Other	Present	Absent	Present	Present	1346849..1360536	NCTC1
Uncharacterised island	Other	Present	Absent	Absent	Absent	1438229..1442289	NCTC1
Uncharacterised island	ND	Present	Present	Present	Absent	1527448..1533616	301
Uncharacterised island	ND	Present	Present	Present	Absent	2149044..2161115	301
Uncharacterised island	ND	Present	Present	Present	Absent	3845970..3853803	301

ND Not determined
